# The Microwell-mesh: A high-throughput 3D prostate cancer spheroid and drug-testing platform

**DOI:** 10.1038/s41598-017-18050-1

**Published:** 2018-01-10

**Authors:** E. O. Mosaad, K. F. Chambers, K. Futrega, J. A. Clements, M. R. Doran

**Affiliations:** 1 Stem Cell Therapies Laboratory, Queensland University of Technology (QUT), Translational Research Institute (TRI), Brisbane, Australia; 2 Australian Prostate Cancer Research Centre – Queensland (APCRC-Q), Brisbane, Australia; 3 Biochemistry division, Chemistry Department, Faculty of Science, Damietta University, Damietta, Egypt; 4 Quadram Institute Bioscience, Norwich Research Park, Norwich, UK; 5 Mater Research Institute – University of Queensland, Translational Research Institute (TRI), Brisbane, Australia; 6 Australian National Centre for the Public Awareness of Science, Australian National University, Canberra, Australia

## Abstract

Treatment following early diagnosis of Prostate cancer (PCa) is increasingly successful, whilst the treatment of advanced and metastatic PCa remains challenging. A major limitation in the development of new therapies is the prediction of drug efficacy using *in vitro* models. Classic *in vitro* 2-dimensional (2D) cell monolayer cultures are hypersensitive to anti-cancer drugs. As a result, there has been a surge in the development of platforms that enable three dimensional (3D) cultures thought to better replicate natural physiology and better predict drug efficacy. A deficiency associated with most 3D culture systems is that their complexity reduces the number of replicates and combination therapies that can be feasibly evaluated. Herein, we describe the use of a microwell platform that utilises a nylon mesh to retain 3D micro-tumours in discrete microwells; termed the *Microwell-mesh*. The Microwell-mesh enables the manufacture of ~150 micro-tumours per well in a 48-well plate, and response to anti-tumour drugs can be readily quantified. Our results demonstrate that 3D micro-tumours, unlike 2D monolayers, are not hypersensitive to Docetaxel or Abiraterone Acetate, providing a superior platform for the evaluation of sequential drug treatment. In summary, the Microwell-mesh provides an efficient 3D micro-tumour platform for single and sequential drug screening.

## Introduction

Common 2-dimensional (2D) monolayer cell culture generally fails to mimic the complex behaviour of native tissue. 2D culture limits cell-cell interaction, modifies gene expression and limits tissue-like matrix accumulation^1^. From a cancer cell biology perspective, one of the most perturbing factors is the high tensile stress experienced by cells cultured on rigid 2D tissue culture plastic surfaces^2,3^. This stress triggers abnormally high cell proliferation rates, which can directly impact or confound the sensitivity of cells to anti-cancer drugs, which often specifically target proliferating cells^4^.

The merits for the use of 3D spheroids to study drug response in cancer research have long been appreciated. In 2002, Jacks and Weinberg summarized the merits of 3D cultures, and closed with the statement “*Suddenly, the study of cancer cells in two dimensions seems quaint, if not archaic*”^2^. Despite disadvantages, the use of 2D culture in cancer drug screening remains common, as standard 2D tissue culture plates are inexpensive, high throughput fluidics systems are compatible with such plates, and a range of platforms are available to facilitate imaging or characterisation of cultures maintained in standard 2D tissue culture plates. Transition to the widespread use of 3D cultures in cancer research is dependent on the development of efficient culture systems that enable similar high throughput capacities.

A number of recent innovations are enabling improvements in the efficiencies of establishing and characterising 3D cultures. Methods for establishing 3D cultures include the use of hydrogel matrices such as Matrigel, or scaffold-free systems including hanging drop culture systems, microfluidic platforms, and microwell platforms^5,6^. The advantage of the later three, relative to hydrogel approaches, is that they enable the manufacture of 3D cell spheroids of controlled size and rely on the cells to produce their own endogenous extracellular matrix. Our group previously demonstrated the utility of microwell platforms to manufacture hundreds of uniform 3D micro-tumours from prostate cancer (PCa) cells and subsequently characterised their response to anti-cancer drugs^3^. A strength of microwell platforms is their decrease in the metabolic activity compared of uniform cell spheroids in a single well^7^.

In our previous work, we utilised a microwell platform fabricated from polydimethylsiloxane (PDMS), with an array of approximately 150 microwells/cm^2^ ^2,3^. This microwell platform, like others previously published in the literature^8–10^, enabled the uniform distribution of cells into microwells at the bottom of a culture well plate by brief centrifugation. Cells in discrete microwells will aggregate to form 3D micro-tumours that can then be permitted to grow in culture and/or be treated with drug(s). A major limitation of microwell platforms, such as the one used in our previous study, is that the addition of drug(s) or the exchange of medium can easily displace micro-tumours from their discrete microwells. Displaced micro-tumours can be lost through medium exchange or fall into adjacent microwells where they can amalgamate with other micro-tumours, resulting in a culture of heterogeneous micro-tumour sizes and numbers. To overcome this limitation our group developed the “Microwell-mesh”^11^. The unique feature of the Microwell-mesh is that it has a nylon mesh with a 36 μm pore size fixed over the microwells. The openings in the mesh are large enough to allow a single cell suspension to pass through and aggregate at the bottom of each microwell, while small enough to prevent aggregated micro-tumours from escaping individual microwells. This feature enables the simultaneous and efficient manufacture of hundreds of uniform micro-tumours, in a format that facilitates the multiple medium exchanges required for complex and/or sequential drug treatment.

In our previous work we utilised the Microwell-mesh to manufacture spheroids of cartilage tissue^11^. Herein, we describe the fabrication and use of a Microwell-mesh platform with smaller microwells, tailored for the formation of micro-tumours and anticancer drug testing. We demonstrate that the Microwell-mesh can be used to establish micro-tumours from defined numbers of PCa cells, and that micro-tumour growth and regression in response to drug treatment can be quantified using methods compatible with high throughput screening assays. Specifically, we characterise the formation and growth of micro-tumours assembled from C42B and LNCaP PCa cells, and micro-tissues assembled from prostate stromal myofibroblasts WPMY-1 cells. We explore the micro-tumour response to two drugs commonly used for the treatment of advanced PCa; treatments evaluated here include androgen targeted therapy, Abiraterone Acetate or Enzalutamide, and single or sequential doses of the taxane chemotherapy, Docetaxel.

## Materials and Methods

### Cell lines and standard cell culture

All cell lines were obtained from the American Type Tissue Collection (ATCC) and included the androgen-responsive C42B and LNCaP cells and prostate myofibroblasts WPMY-1 cells. Cell lines were authenticated at the Genomic Research Centre (GRC; Brisbane, Australia) using Short Tandem Repeat (STR) analysis. STR profiles of the cell lines were compared to the ATCC STR Database to verify cell line identity; and all cell lines showed ≥80% match to the corresponding reference STR profile. Cells were cultured in low glucose Dulbecco’s modified Eagle’s medium (DMEM-LG) supplemented with 10% fetal bovine serum (FBS; Invitrogen) and 1% penicillin/streptomycin (Invitrogen). For some assays, FBS was replaced with 10% charcoal stripped fetal bovine serum (CSS; Invitrogen) to mimic androgen deprivation conditions. Cells were grown in a cell culture incubator at 37 °C and 5% CO_2_. All cells were passaged when monolayers reached ~80% confluency using 0.25% Trypsin/EDTA (Invitrogen).

### Microwell-mesh design and fabrication

The fabrication of microwell arrays of polydimethylsiloxane (PDMS) was performed as described previously^3,11^. Briefly, a polystyrene mold was used to cast arrays of approximately 150 microwells/cm^2^. Each microwell was 800 × 800 μm square, by 500 μm deep. Liquid PDMS was poured onto polystyrene molds to form 2 mm thick sheets and cured at 60 °C for 1 hour. Discs, 11 mm in diameter, were punched out of the PDMS sheets using a wad punch (Amazon). Disc size was selected such that these discs would fit snuggly into 48-well plates (Nunc, Thermo Fisher Scientific). Nylon mesh (36 µm^2^ pore openings, part number: CMN-0035, Amazon) was fixed to the top of the microwells using silicone glue (Selleys, Australia). Once the glue had cured, excess mesh was trimmed from the disc inserts using scissors. Inserts were then anchored into individual wells in 48-well plates by placing a small amount of silicone glue at the bottom of the well, and the insert pressed into the well. Plates with Microwell-mesh inserts were sterilized in 70% ethanol for 1 hour, followed by several (3–4) washes with Dulbecco’s phosphate buffered saline (DPBS), and stored wet in DPBS until use. To prevent cell adhesion to the PDMS surface, wells with Microwell-mesh inserts were treated with 0.25 mL of sterile 5% Pluronic-F127 (Sigma), which was centrifuged at 1,000 *g* to penetrate into each microwell, and permitted to adsorb to the PDMS surface for >10 minutes^7^. Treated surfaces were washed twice with DPBS prior to cell seeding.

### Cell seeding and culture in the Microwell-mesh

In this study, we aimed to form micro-tumours (cancer cells) and micro-tissues (non-cancer cells) from 600 cells each. Inserts had approximately 150 microwells each, and so single cell suspensions containing 90,000 cells in 0.5 mL of medium were seeded into each well of 48-well plate. Plates were then centrifuged at 400 × g for 5 minutes to force cells through the mesh and aggregate the cells uniformly at the bottom of each microwell. Standard 2D culture controls were established by seeding cells at 10,000 cells/cm^2^. The aggregation of cells into microwells was visually confirmed using an Olympus CKX14 microscope, and images captured using an Olympus DP26 digital camera (Japan) and Microscopy software (CKX14, CellSens Entry). Plates were then transferred to a cell culture incubator maintained at 37 °C and 5% CO_2_.

Cultures were maintained for up to 14 days. A half-volume (0.25 mL) culture medium exchange was performed every second day. Images were captured every two days for diameter measurement. A minimum of 50 micro-tumours formed from C42B or LNCaP cells and micro-tissues formed from WPMY-1 cells were measured per time point. Four replicate cultures were harvested every second day for DNA quantification or at day 1 and 7 for immunofluorescent staining.

### Immunofluorescence staining and confocal imaging

Spheroids were harvested by peeling the nylon mesh from the microwells, and collecting the spheroids into Eppendorf tubes. Spheroids were fixed using 4% PFA for 30 minutes at room temperature, followed by permeabilisation using 0.5% Triton X-100 in DPBS for 30 minutes at room temperature. To prevent non-specific binding, 5% bovine serum albumin (Sigma, A7906) was used in the blocking step for 1 hour at room temperature. Cell aggregates were then incubated with primary antibody for Ki67 (Abcam, ab92742) at 1  µg/ml overnight at 4 °C. The anti-rabbit secondary antibody conjugated with Alexa Fluor 594 (Invitrogen; dilution 1:500) was added to the aggregates for 1 hour at room temperature, followed by the nuclear stain, 4′,6-diamidino-2-phenylindole (DAPI, Sigma-Aldrich), for 30 minutes at room temperature. Stained spheroids were imaged using a Zeiss 510 Meta confocal microscope.

### Drug testing in cell cultures

Docetaxel (Sigma, 01885), Abiraterone Acetate (Sigma, SML 1527) and Enzalutamide (Haoyuan Chemexpress, HY-70002) were purchased as powders and dissolved in Dimethyl sulfoxide (DMSO; Sigma-Aldrich, 472301), then aliquoted and stored at −80 °C. On the day of treatment, an aliquot was thawed and diluted to the indicated concentrations using culture media. Before selecting the culture densities used in drug testing experiments, multiple cell densities were tested, specifically 5000, 25,000 and 45,000 cells/cm^2^ in 2D cultures and 150, 300 and 600 cells/micro-tumour in 3D cultures. The impact of prolonged culture period prior to single Docetaxel treatment was also tested.

**Figure 1:**
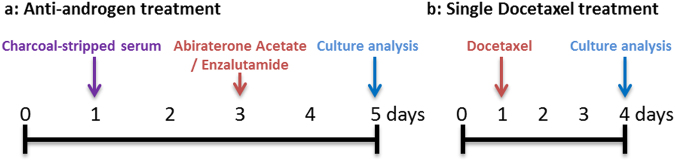
Drug treatment protocol. (**a**) For androgen deprivation, cell cultures where seeded in standard FBS supplemented medium on day 0 and replaced with CSS medium 24 hours later (day 1) to starve cells of androgen. On day 3, Abiraterone Acetate or Enzalutamide was added to cell cultures. Cultures were terminated on day 5 and analysed using the specified methods. (**b**) A single treatment of Docetaxel was performed after 24 hours of seeding the cells (day 1). Drug response was then assessed following three days of exposure to Docetaxel (day 4).

For drug testing experiments, cells were seeded in 48 well plates at 10,000 cells/cm^2^ in 2D cultures and 600 cells/micro-tumour in 3D cultures. All cells were cultured overnight to permit plastic adherence or self-aggregation in 2D and 3D cultures, respectively. The treatment protocols used to evaluate the anti-tumour drugs are illustrated schematically in the text adjacent to the relevant experimental data sets. For anti-androgen treatment (Fig. 1 a), cultures were first initiated in medium containing 10% FBS (day 0) and permitted to stabilise overnight. The next day (day 1), culture media were replaced with fresh culture medium supplemented with 10% CSS to mimic androgen deprivation conditions for 48 hours. On day 3, culture medium was replaced with fresh 10% CSS medium containing Abiraterone Acetate or Enzalutamide and cultures were incubated for a further 48 hours. Following this period (on day 5), cultures were assessed for metabolic activity, as well as ATP and DNA content. For single cytotoxic drug treatment experiments (Fig. 1b and Supplementary Figure 1), cultures were established overnight or for 3 days in 10% FBS and then treated with medium containing 10% FBS and Docetaxel for 72 hours.

In experiments where cells were exposed to sequential Docetaxel treatment, the cultures were permitted to recover from the first Docetaxel treatment for 72 hours in drug-free medium supplemented with 10% FBS. Following this recovery period, cells were exposed to a second 72-hours period of Docetaxel treatment. Metabolic activity and DNA content of Docetaxel-treated cells were quantified at day 4 for single dose-treated cultures and at day 4, 5, 7, 10 and 11 for sequential dose-treated cultures.

### Metabolic activity measurement

AlamarBlue is a metabolic assay commonly used as an indirect method to estimate viable cell numbers. The AlamarBlue assay consists of a non-fluorescent blue dye (Resazurin), which is reduced to a fluorescent pink compound (Resorufin) by the action of mitochondrial and cytoplasmic reductases of living cells^12^.

One of our objectives was to determine whether the AlamarBlue assay could be used by direct addition and incubation of the AlamarBlue reagent in the Microwell-mesh cultures and subsequently assessed by placing the entire plate in a fluorescence plate reader. To optimise the AlamarBlue assay, several concentrations of the assay reagent (3, 5 and 10% of the culture volume) were added to the culture media of increasing number of PCa cells in 2D monolayers and 3D Microwell-mesh cultures. Plates were incubated at 37 °C for 1 to 5 hours. Data was acquired by two different settings of the spectrophotometer plate reader, where the fluorescence measurement was collected from the top or the bottom of the plate. The fluorescence was detected using a FLUOstar Omega Microplate Reader (BMG LABTECH) with a 544 nm excitation and 590 nm emission. The fluorescence values were plotted against cell numbers to determine the linearity of the assay and to identify the optimum reagent concentration and incubation time for performing the assay *in situ* (directly in the Microwell-mesh plate).

### ATP measurement

The amount of ATP in the 2D or 3D Abiraterone Acetate treated cultures was measured using the CellTitre-Glo 3D Cell Viability Assay kit (Promega) according to the manufacturer’s instructions. This assay relies on the addition of exogenous thermostable luciferase enzyme that generates a luminescence signal from the conversion of D-luciferin in the presence of cellular ATP. Similar to the AlamarBlue assay, quantification of ATP in cell lysates is often used as an indirect estimate of the number of viable cells. At the specified time points, half of the culture media volume was removed and replaced with the ready-to-use cell lysis reagent. To facilitate lysis, plates were placed on an orbital plate shaker for 5 minutes, and then incubated for a further 20 minutes at room temperature. Samples were collected and bioluminescence was measured using a PHERAstar FS plate reader (BMG LABTECH). A standard curve was generated using ATP disodium salt (Sigma, A7699). Results are represented as values normalised to the amount of ATP produced in the control cultures (10% FBS-supplemented media conditions).

### DNA quantification

DNA quantification was performed as another means to estimate cell number. The Quant-iT PicoGreen dsDNA assay (Invitrogen) was used as per manufacturer’s instructions to fluorescently measure the double stranded DNA content contained in cell cultures. To lyse cell monolayers and spheroids, two freeze/thaw cycles were performed in TE buffer (20 mM Tris-HCl, 2 mM EDTA, pH 7.5) containing 1% Tween 20. Cell lysates were mixed with PicoGreen reagent and fluorescence read using 485 nm excitation and 520 nm emission (FLUOstar Omega Microplate Reader; BMG LABTECH). DNA content was calculated using a standard curve of ʎ DNA as a reference. Results are represented as values normalised to the amount of DNA in the control cultures (10% FBS-supplemented media conditions).

### Cellular oxidative activity assessment

Dihydrorhodamine 123 (DHR123) was utilised to quantify reactive oxygen species within the LNCaP cells cultured in androgen-deprived conditions. Following drug treatment as described above (Fig. 1 a), cells were washed to eliminate remaining drug then incubated with 1 µg/ml DHR123 for 1 hour at 37 °C, followed by two wash steps with DPBS. Cells were then trypsinized, resuspended in MACs buffer (Miltenyi Biotech), and analysed on a BD LSRII flow cytometer (Becton Dickinson). Data was analysed using FlowJo software (TreeStar, USA). The mean fluorescence values of four individual samples were calculated and represented in the results section following normalization against control culture (10% CSS- supplemented media conditions).

### Statistical analysis

Results represent the mean values of 3 independent experiments, each performed with 4 biological replicate cultures, unless mentioned otherwise. Error bars represent standard deviations. Statistical significance of data was evaluated using one-way or two-way analysis of variance (ANOVA), where specified, using Prism software, Version 6.0 (GraphPad). P-values for each comparison are represented by asterisks as indicated in figure captions.

## Results

### The Microwell-mesh retains micro-tumours in discrete microwells

**Figure 2:**
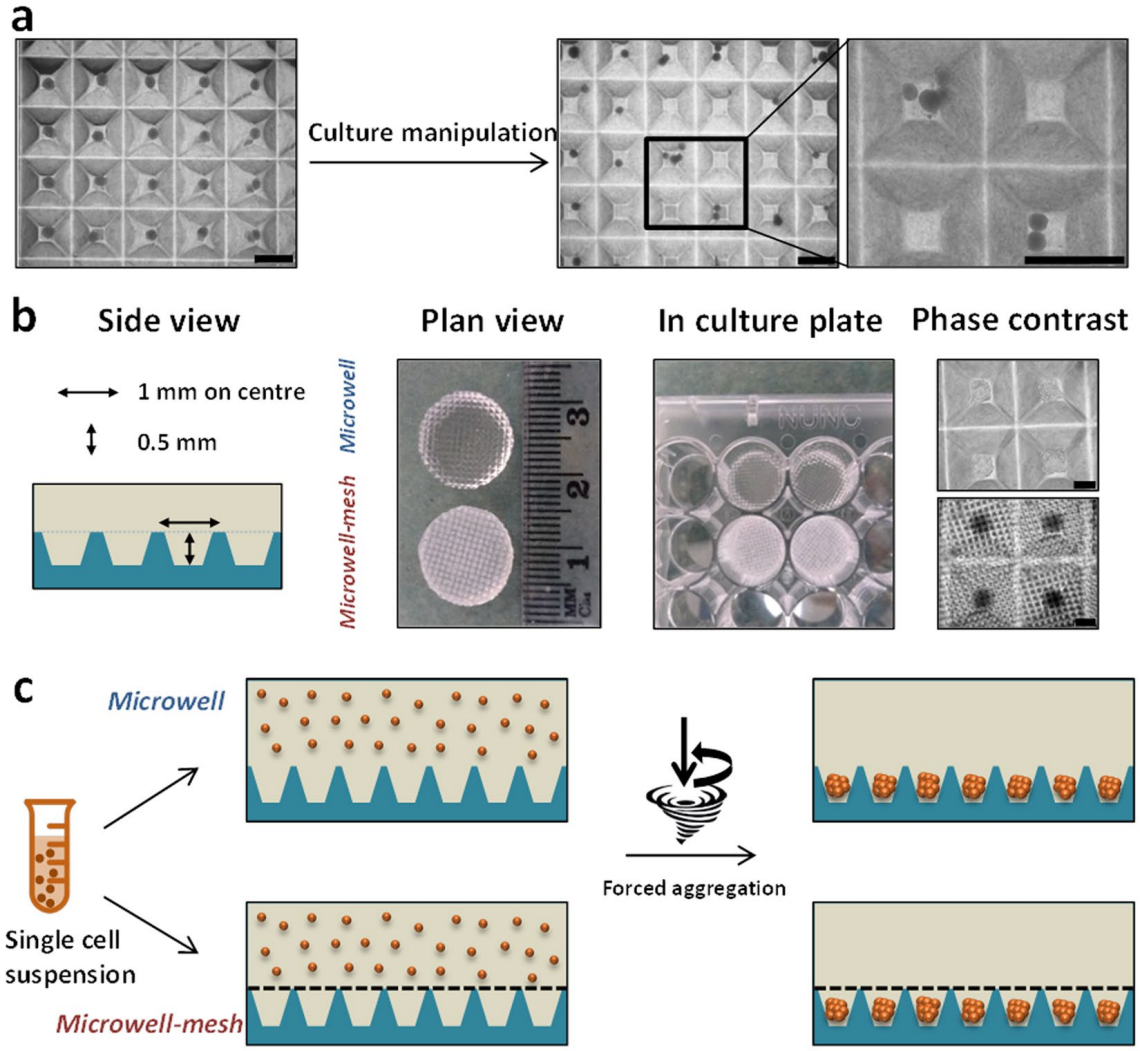
Microwell platform manufacture and establishment of 3D micro-spheroid culture. (**a**) The traditional open-top microwell platform results in dislodged spheroids following culture media exchange (scale bar = 500 µm). (**b**) Schematic illustration and bright field images show PDMS discs with and without the mesh which can be inserted in 48-well tissue culture plates and cells aggregated after 24 hours of seeding cell suspension (scale bar = 200 µm). (**c**) Schematic illustration of cell seeding using traditional open-top microwells (top) and Microwell-mesh being microwells modified with a 36 µm mesh-top (bottom) large enough to allow single cells to pass through, but small enough to retain spheroids within discrete microwells.

A central goal of our work was to create a microwell platform and method that would ultimately lend itself to complex sequential drug testing. We trialled traditional open top microwell platforms, but found that micro-tumours were often displaced from their discrete microwells, and randomly accumulated in adjacent wells (Fig. 2a). Based on these results we fabricated the Microwell-mesh platform shown in Fig. 2b and c. The Microwell-mesh platform enabled retention of multicellular micro-tumours in discrete microwells during sequential medium and drug exchange.

### Cell numbers can be assessed in the Microwell-mesh using AlamarBlue

**Figure 3:**
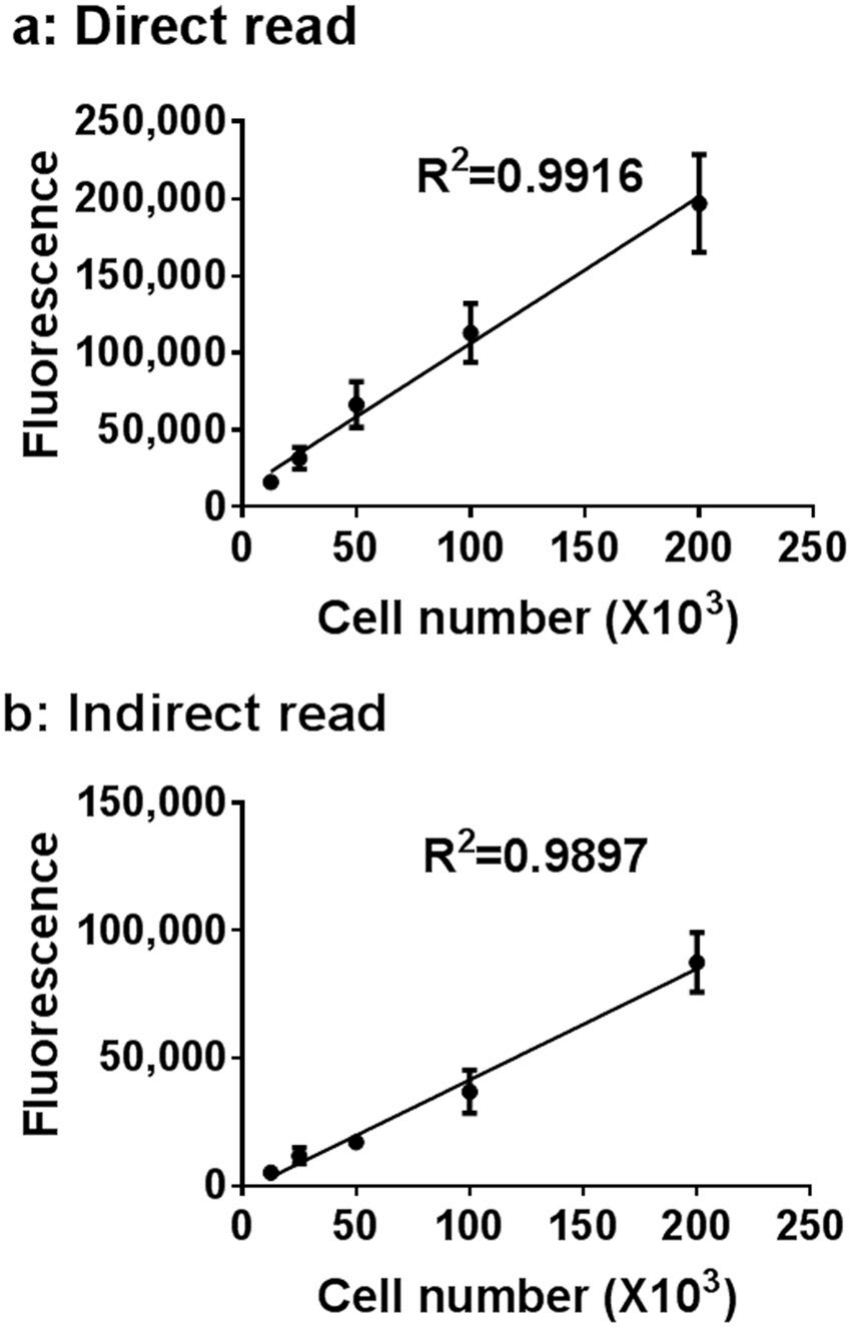
AlamarBlue assay optimization in Microwell plates. No significant change in the linearity of the AlamarBlue assay after 3 hours incubation with 3% of AlamarBlue reagent when the fluorescence was acquired *in situ* (**a**) or when the reaction product was transferred to a black 96-well plate (**b**).

To ensure that the Microwell-mesh insert did not interfere with assessing the AlamarBlue assay in 3D cultures, we measured the fluorescence of the assay directly in the plate well, and following transfer of the reacted media to a new well plate (containing no Microwell-mesh insert). To optimise the AlamarBlue assay in 3D Microwell-mesh cultures, titrations of cell numbers were prepared to generate standard curves and also compared to 2D monolayer cultures (Supplementary Figure 2). Measurements were taken at 1, 2, 3, 4 and 5 hours at 3%, 5% and 10% concentrations of AlamarBlue. The cumulative results indicated that the most reliable results could be obtained when measurements were performed with 3% AlamarBlue reagent and incubated for 2 and 3 hours in 2D and 3D cultures, respectively. We trialled reading the AlamarBlue fluorescence signal either by transferring portions of the medium to black 96-well plates, or reading the fluorescence signal directly from the Microwell-mesh plate either from the bottom or top of the plate (Fig. 3 and Supplementary Figure 2). All modes of measurement generated similar, linear standard curves. This result indicated that neither the PDMS insert nor the clear tissue culture wells compromised the sensitivity of the AlamarBlue assay, and indicated that *in situ* measurement of AlamarBlue fluorescence was a suitable strategy to quantify relative cell numbers directly from the Microwell-mesh plates.

### PCa 3D micro-tumour growth

**Figure 4:**
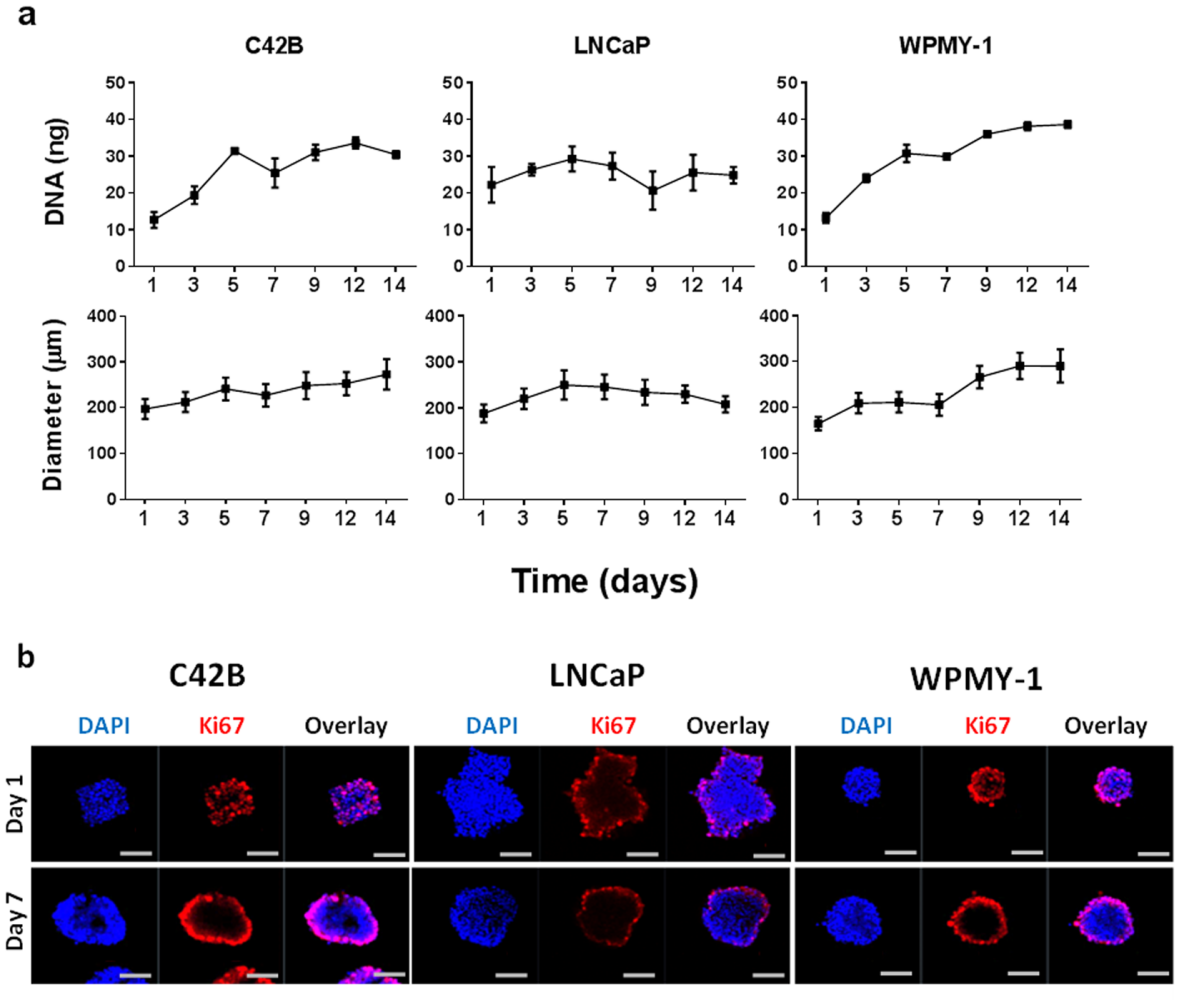
Characterisation of prostate cancer cell lines in 3D micro-tumour culture. (**a**) Prostate cancer (C42B and LNCaP) and prostate myofibroblasts (WPMY-1) cell lines were cultured in the 3D platform (600 cells/spheroid) and the growth of cell spheroids was assessed by DNA quantification and spheroid diameter measurement. DNA quantification data represents the mean value of four replicate cultures. A minimum of 50 spheroids were measured per time point for diameter measurement. (**b**) Confocal images of spheroids (600 cells/spheroid) stained with Ki67 (red) and nuclear stain (DAPI; blue) were acquired on day 1 and 7 of culture. Scale bar = 100 µm.

We quantified the growth of C42B and LNCaP micro-tumours and WPMY-1 micro-tissues each initiated from 600 cells over culture periods of 14 days with a half-volume culture medium exchange every second day. Micro-tumours generated from both PCa cell lines increased in diameter with a corresponding increase in DNA quantity over the first 5–7 days, after which proliferation rates gradually plateaued (Fig. 4a). By contrast, micro-tissues formed from prostate myofibroblasts (WPMY-1) continued to proliferate over 14 days as evidenced by increasing DNA quantity and spheroid diameter (Fig. 4a). Bright field images showed the three-dimensionality of the uniform micro-tumours/micro-tissues generated from the three cell lines under examination in the microwell platform. Therefore, the volume of the micro-tumours/micro-tissues were calculated using the diameter measurements over time following the formula V = 4/3 π r^3^, where V is the volume and r is the radius of the sphere (Supplementary Figure 3).

Ki67 staining was used to identify proliferating cells in 3D micro-tumour cultures. Confocal images of the micro-tumours revealed that Ki67 positive cells were evenly distributed throughout the spheroids at day 1, but by day 7 most proliferating cells were localised to the outer surface of the micro-tumours (Fig. 4b). Similar to the micro-tumours, WPMY-1 micro-tissues showed the same distribution of Ki67 positive cells at day 1 and day 7 of culture.

### 3D micro-tumours of PCa cells are less sensitive to conditions that replicate androgen deprivation

Androgens are a critical player in prostate cancer cell growth and progression to advanced stage castrate resistant PCa results in the cells becoming resistant to anti-androgen treatment^13^. Therefore, we hypothesized that our 3D culture system would better reflect the response to androgen deprivation compared to 2D monolayer cultures. To test this hypothesis, we cultured PCa cell lines in an androgen deprived setting (CSS- supplemented culture media), and then treated the cells with Abiraterone Acetate, a first-in-class inhibitor of the CYP17A enzyme to prevent the biosynthesis of androgens intracellularly from their steroidal precursor^14^ and used three different assays to assess the cellular responses in our 2D and 3D cultures.

**Figure 5:**
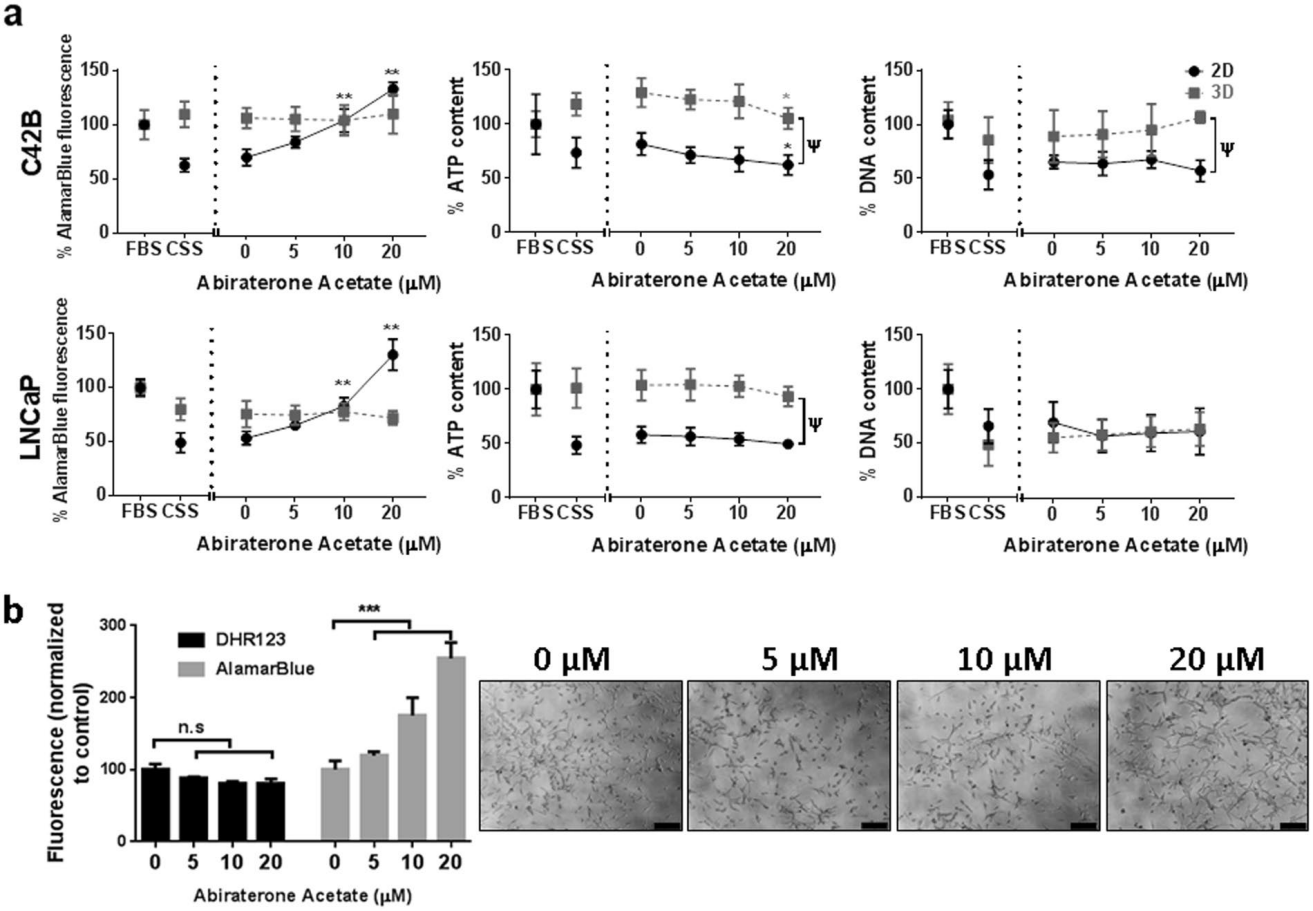
Monolayer and micro-tumour behaviour of C42B and LNCaP cell lines in androgen deprived conditions. (**a**) C42B (Top) and LNCaP (Bottom) cells were seeded in expansion culture medium for 24 hours followed by medium exchange to androgen-depleted medium (CSS) for a further 48 hours. Abiraterone Acetate was then added to the culture medium at the indicated concentrations for an additional 48 hours. AlamarBlue, Cell Titre-Glo 3D Cell Viability and PicoGreen assays were then performed to assess metabolic activity, ATP quantity, and DNA quantity, respectively. All results are represented as a percentage of the FBS-containing culture medium control values (data was collected from three independent experiments, each having four technical replicate cultures). Statistical significance was calculated by two-way ANOVA compared to the corresponding zero value (**P < 0.0001 and *P < 0.01) or compare 2D and 3D values with the same drug treatment (Ψ P < 0.005). (**b**) Metabolic activity (AlamarBlue assay) and DHR123 staining of LNCaP monolayers at specified Abiraterone Acetate concentrations. Results represented as the mean fluorescence values of four individual samples normalized to control culture values. Statistical significance was performed using one-way ANOVA (*** P<0.001). Side panel represents the cellular morphology of LNCaP cells at the indicated Abiraterone Acetate concentrations (µM). Scale bar = 200 µm.

In 2D monolayers, PCa cell lines (C42B and LNCaP) cultured in androgen-depleted media (CSS) showed a reduction in metabolic activity, as assessed by the AlamarBlue assay, and a reduction in ATP and DNA quantity, compared to androgen-replete media (FBS) (Fig. 5a). A 48-hour treatment of 2D PCa cultures with increasing concentrations of Abiraterone Acetate (0–20 µM) resulted in an unexpected increase in metabolic activity (AlamarBlue assay) and this was dose-responsive in both cell lines (P < 0.0001 for 10 and 20 µM). In contrast, cellular ATP concentrations were unchanged at lower Abiraterone Acetate concentrations (5 and 10 µM) in 2D PCa cell cultures and slightly reduced at the highest concentration (20 µM) in only the C42B cultures (P < 0.01), relative to control cultures in CSS plus vehicle (0 µM). DNA concentrations were unchanged in response to Abiraterone Acetate in both PCa cell lines, compared to control. Similarly, LNCaP cells monolayers treated with Enzalutamide instead of Abiraterone Acetate exhibited an unexpected dose-dependent increase in AlamarBlue readout. While the AlamarBlue readout increased with increasing doses of Enzalutamide, ATP concentrations were unchanged and DNA concentrations slightly decreased with higher Enzalutamide concentrations (1 µM and 20 µM) (Supplementary Figure 4). We continued to evaluate the response of PCa cells to anti-androgen in 3D micro-tumour culture, but restricted our focus to characterising cell response to Abiraterone Acetate.

In 3D micro-tumour cultures, metabolic activity and ATP quantity was not significantly different between androgen-depleted and androgen-replete cultures for both PCa cell lines (Fig. 5a). In contrast, a significant drop in DNA quantity was observed in 3D cultures under androgen-depleted conditions. The addition of increasing concentrations of Abiraterone Acetate to 3D cultures (0–20 µM) did not result in changes in metabolic activity, ATP quantity or DNA quantity, except for in C42B cultures, where the ATP concentration was slightly reduced at 20 µM Abiraterone Acetate (P < 0.01). Overall, 3D PCa cultures were much less responsive to both androgen-depletion and Abiraterone Acetate treatment, relative to 2D cultures.

To determine whether the increased metabolic activity, measured by AlamarBlue assay, in Abiraterone Acetate-treated 2D cultures was due to an increase in the mitochondrial redox activity, we tested the mitochondrial redox activity of drug treated LNCaP cells (Fig. 5b). Fluorescence intensities of vehicle or DHR123 treated cells were analysed by flow cytometry. Results showed no significance change in internal cell redox in Abiraterone Acetate-treated cells compared to untreated cells, while a significant increase in metabolic activity measured by AlamarBlue assay was shown in a parallel experiment. We interpreted this as an indication that mitochondrial activity was likely not upregulated in the Abiraterone Acetate-treated cells. The bright field images revealed no significant change in the cellular morphology of low dose (0–10 µM) Abiraterone Acetate-treated LNCaP cells compared to the control, except in the high dose of Abiraterone Acetate (20 µM) where the cells showed more irregular morphology.

It is important to mention that we observed no change in AlamarBlue fluorescence in cell-free cultures treated with Abiraterone Acetate (Supplementary Figure 5) which confirms cellular participation in the reduction of the AlamarBlue and not a direct reduction by the drug.

### 2D and 3D PCa culture response to Docetaxel

**Figure 6:**
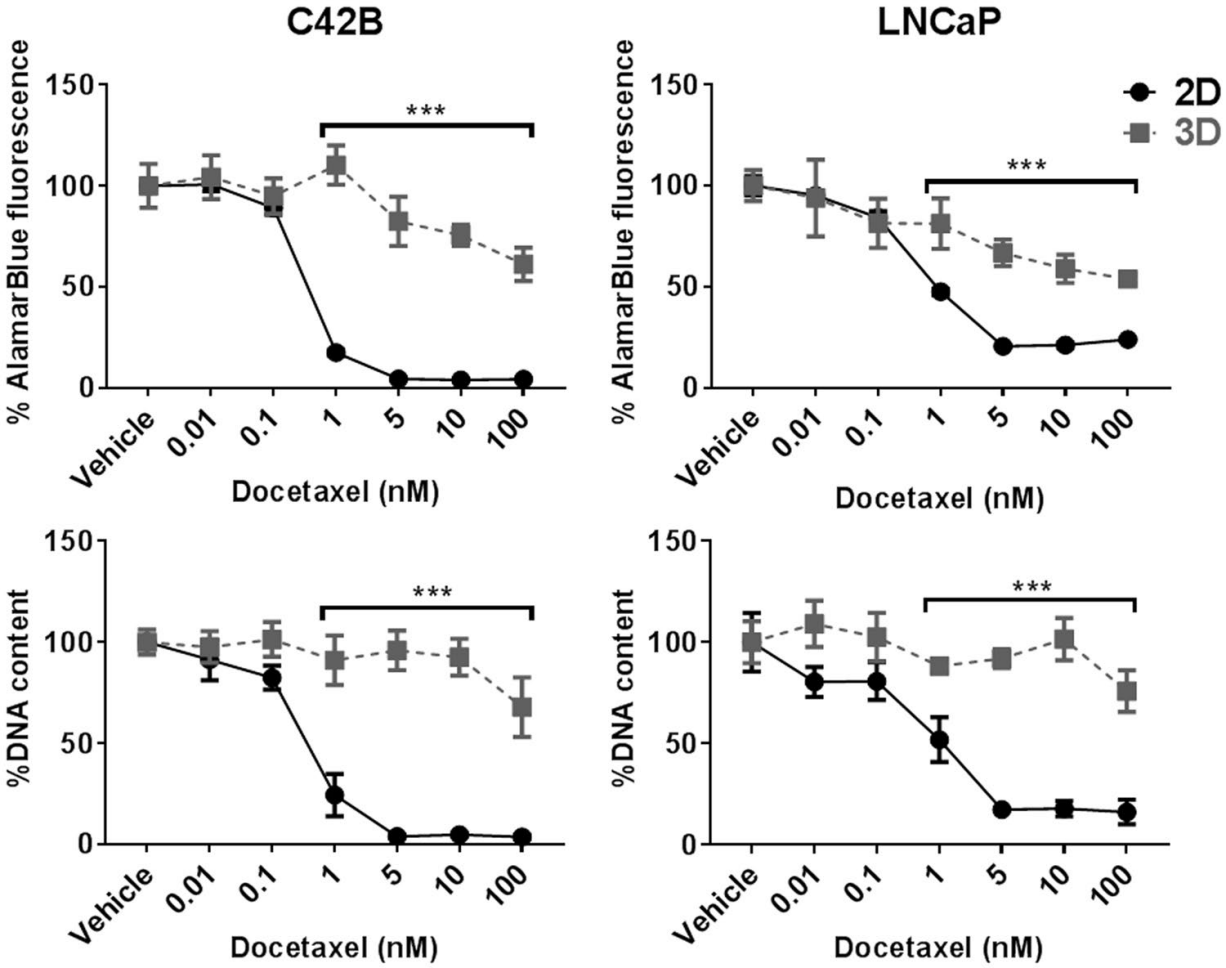
C42Band LNCaP Docetaxel drug response. C42B and LNCaP cells in 2D and 3D cultures were treated with Docetaxel in the indicated concentrations for 72 hours followed by metabolic activity and DNA content measurements. All results are represented as a percentage of the vehicle control values (data was collected from three independent experiments each had four replicate cultures n = 4). Statistical significance was calculated by two-way ANOVA compared to the corresponding values in 2D cultures (***P < 0.0001).

Next, we evaluated the response of PCa cell lines in 2D and 3D cultures to a single dose of Docetaxel at varying concentrations in the range of 0 nM (Vehicle only) to 100 nM in expansion media (Fig. 6). In 2D cultures, both C42B and LNCaP cells showed a significant drop in metabolic activity and DNA quantity at 1 nM Docetaxel, which was maximally reduced by 5 nM Docetaxel. While the metabolic activity of both cell lines slightly decreased in Docetaxel-treated 3D cultures, significant differences in metabolic activity and DNA content between 2D and 3D cultures were observed in cultures treated with ≥1 nM Docetaxel. In this study, the IC50 values of Docetaxel in 3D cultures (>120 nM and 24 nM for C42B and LNCaP cells, respectively) were considerably elevated relative to IC50 values in 2D cultures (approximately 0.4 nM and 1 nM for C42B and LNCaP cells, respectively).

As the density of the cell culture is a critical factor in drug screening platforms, we compared drug responses of short (1 day) and long (3 days) pre-treatment cultures. Interestingly, 2D and 3D cultures treated with Docetaxel after 1 day of establishing the cultures showed higher sensitivity towards Docetaxel treatment. However, micro-tumours cultured for prolonged periods (3 days) prior to Docetaxel treatment showed reduced sensitivity against the drug compared to corresponding 2D monolayer cultures (Supplementary Figure 6).

### Cellular recovery after sequential cytotoxic drug treatment is enhanced in 3D cultures

One of the many advantages of our 3D culture platform is that it permits several culture manipulations without displacement or loss of cellular spheroids from discrete microwells. This improvement enables testing metronomic or sequential treatments of one or several drugs for the preclinical studies.

**Figure 7:**
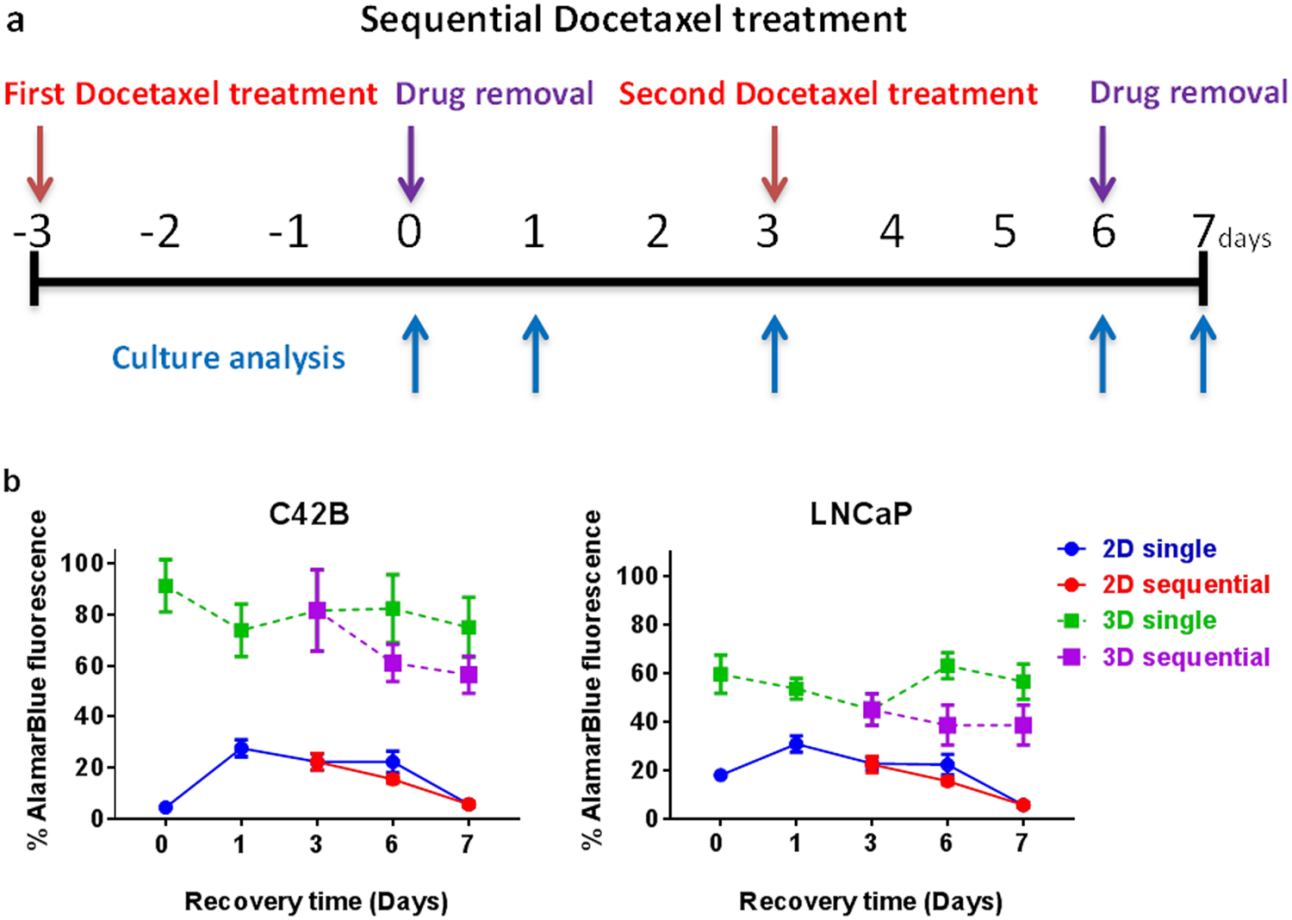
Sequential Docetaxel treatment and prostate cancer cell recovery. (**a**) Sequential treatment of Docetaxel was performed at the indicated time points (red arrows). Each treatment for 72 hours was followed by metabolic activity measurement (blue arrows) and drug removal (purple arrows) at day zero and 6. In addition, cell recovery assessment was also performed at days 1, 3, 6 and 7 for single treatments and at days 6 and 7 for sequential treatments. (**b**) 2D and 3D cultures of C42B and LNCaP cells were treated with 5 nM Docetaxel following the sequential treatment protocol and the metabolic activity of the survived cells were assessed at the indicated time points using AlamarBlue assay. All results are represented as a percentage of the vehicle control values (Two independent experiments each had four replicate cultures n = 4).

We assessed the metabolic activity of PCa cells cultured in monolayers or 3D micro-tumours and treated with 5 nM Docetaxel in sequential doses (Fig. 7a). Despite the slight decline in metabolic activity of single dose-treated 3D micro-tumours after the first day of drug-free culture, the metabolic activity of 3D micro-tumours was gradually restored by day 6 of culture. Following the second Docetaxel treatment at day 3, 3D micro-tumours demonstrated a slight decrease in the metabolic activity compared to single dose-treated cultures. LNCaP micro-tissues demonstrated greater sensitivity towards Docetaxel compared to C42B micro-tissues.

By contrast, PCa monolayers continued to suffer a decline in metabolic activity despite a transient increase in activity during the first day of culture following the first drug treatment. After 7 days of culture, both single and sequential Docetaxel-treated 2D monolayer cultures of C42B and LNCaP cells showed equal metabolic activities (<10% compared to vehicle treated cultures). In general, cells grown in 3D cultures were metabolically more active than that in 2D cultures either after single or sequential Docetaxel treatments.

## Discussion

3D tissue culture systems have gained popularity in recent years. However, widespread adoption of 3D models remains low. This is due to limitations associated with 3D culture systems including heterogeneity of cultured spheroid sizes, or heterogeneity within single tissues when large macroscopic tissues are used^15,16^. Additionally, the low throughput nature of some 3D tissue manufacturing approaches and the incompatibility of these 3D tissues with high-throughput imaging and other analytical tools has limited uptake. In seeking to engineer a powerful 3D culture system capable of enabling both high-throughput 3D micro-tumour formation and complex drug/medium manipulation, we aimed to improve on microwell platforms commonly used to generate 3D tissue models^11^. Our improvement was the inclusion of a nylon mesh bonded over the microwells (the Microwell-mesh) in such a way that would allow the retention of individual micro-tumours in discrete microwells over multiple culture medium manipulations, enabling complex sequential drug treatment and analysis (Fig. 2). This platform retains the original significant advantage of other microwell systems, enabling the rapid manufacture of hundreds of replicate uniform sized micro-tumours, assembled in a way that allows precise control of the number of cells used to form each individual micro-tumour^3,17–19^.

Using the microwell platform, PCa micro-tumours grew in size over 14 days of culture (Fig. 4a). The increase of cellular content and spheroid size was cell line-dependent. However, a decrease in proliferation over time was observed in all cell lines tested. Our group and others have reported similar observations previously^3,20,21^. Cell proliferation in 2D is often greater than in 3D spheroids because the increase in cell tension experienced by cells cultured on ultra-stiff 2D surfaces stimulates proliferation^22^. Cells in self-assembled spheroids do not experience similar tensile forces, and proliferation rates in spheroids are generally low^23^. Cells on the surface of the spheroids experience the greatest tension, where they can spread over the outside of the spheroid on a flat, albeit curved, surface^24^. Corresponding to this model, we observed that cell proliferation was largely localised to the surface of individual spheroids from days 1–7 for all cell types tested (Fig. 4b).

As the cell spheroid grows, chemical and oxygen diffusion gradients develop across the radius of individual spheroids. These gradients can influence cellular proliferation and viability^25–27^. In cases where spheroids are very large, nutrient supply to the centre of the spheroid can be limiting, leading to the formation of a necrotic core^21,28^. This is commonly reported in spheroids with sizes >500 µm in diameter^16^. In our study, each spheroid was formed from 600 cells with size <300 µm after 14 days of culture, and we did not observe formation of a necrotic core suggesting that metabolite limitation was not a problem in our micro-tumour models. If a hypoxic or necrotic micro-tumour core was desired, this could easily be integrated into the model simply by increasing the number of cells per spheroid. A benefit of the Microwell-mesh platform is that it would enable formation of multiple micro-tumours of similar sizes to facilitate such analysis. If very large micro-tumours are desired (up to ~1.5 mm diameter), then our previously reported larger microwell platform would be more suitable than the smaller version described here^11^.

A major focus of the PCa field is the identification of combination and sequential drug regimens that more effectively treat metastatic and/or castrate-resistant prostate cancer (CRPC)^29,30^. Treatment with Abiraterone Acetate, a potent selective inhibitor of CYP17A, can enhance survival in metastatic CRPC patients^31^. Similarly, Docetaxel is commonly used to treat advanced PCa patients^13^. Resistance to one or both is common, and the capacity to predict the optimal sequence or combination of these drugs (including with other existing or novel drugs) using *in vitro* model systems is a major goal in the field. For the reasons described above, it is believed that 3D models are likely to be more effective tools enabling *in vitro* prediction of drug efficacy.

In the study described here we characterised 3D micro-tumour response to two common PCa drugs, Abiraterone Acetate and Docetaxel. We considered both single and sequential treatment outcomes, and compared the results to 2D PCa cell culture controls. A primary goal of our study was to develop methods that would enable the high-throughput characterisation of PCa micro-tumours within the Microwell-mesh platform; we view the capacity for high throughput analysis to be a major obstacle in the widespread uptake of 3D culture as a preferred tool for *in vitro* drug screening studies.

Our results demonstrated that AlamarBlue could be reliably used to indirectly predict viable cell number in the Microwell-mesh plate simply by adding reagent to the plate, incubating the culture with the AlamarBlue and reading the signal from the plate (Fig. 3). The presence of the PDMS microwell insert and nylon mesh did not confound the reading, and data indicate that future high throughput characterisation of culture drug response could be done directly within Microwell-mesh plates. We subsequently used this approach to characterise C42B and LNCaP micro-tumour sensitivity to Docetaxel. Over a number of experiments, we found that both C42B and LNCaP micro-tumours demonstrated less sensitivity to Docetaxel than control 2D monolayers (Fig. 6). In addition, because the Microwell-mesh retains micro-tumours, we were able to explore cellular response to sequential Docetaxel treatment. Again, 3D micro-tumours displayed reduced hypersensitivity to Docetaxel relative to 2D monolayers. Future studies could use the Microwell-mesh platform to track the evolution of Docetaxel resistant cell populations, or the influence of combinations of drugs and/or different stromal cell populations on treatment outcomes. A critical observation was that the mesh did indeed retain micro-tumours over multiple medium exchanges and wash steps (no cell loss), something not possible when using conventional microwell platforms or 2D cultures.

While AlamarBlue could be used to indirectly estimate viable cell numbers in Docetaxel treated micro-tumour cultures, this was not the case for anti-androgen treated cultures. We were surprised to observe that AlamarBlue conversion (taken as a surrogate for metabolic activity) appeared to increase significantly in cultures treated with Abiraterone Acetate or Enzalutamide (Fig. 5 and Supplementary Figure 4). This apparent increase in metabolic activity, as inferred by the AlamarBlue assay, was particularly evident in 2D control cultures. If taken on its own, the AlamarBlue data suggested that cell numbers were largely unchanged in 3D cultures in response to Abiraterone Acetate, while cell numbers significantly increased in 2D cultures in response to increasing doses of Abiraterone Acetate (Fig. 5a). This would be an unexpected outcome. Reassuringly, microscopy images of 2D treated cultures (Fig. 5b) suggested that there was increasing cell death in response to increasing dose of Abiraterone Acetate. To validate the microscopy observations, we measured ATP concentration and DNA content in 2D and 3D cultures treated with Abiraterone Acetate (Fig. 5a). Both ATP and DNA quantification suggested that 3D cultures were not impacted by Abiraterone Acetate treatment, but that cells in 2D cultures were indeed dying as the images suggested. Multiple replicate experiments demonstrated that there is a robust disconnect between ATP and DNA content relative to AlamarBlue signal evolving from 2D cultures treated with Abiraterone Acetate or Enzalutamide. It is well known that not only mitochondrial enzymes can reduce AlamarBlue reagent to its fluorescent isoform (Resorufin), it can also be reduced by cytoplasmic reductases and the electron transport system of the cell^12^. We reasoned that mitochondrial activity might be upregulated in response to the apoptotic signals generated by the Abiraterone Acetate treated cells^32^, so we used DHR123 and flow cytometry to infer mitochondrial activity in these cells (Fig. 5b). While AlamarBlue conversion was greater in the Abiraterone Acetate treated cells, there was a drop in DHR123 signal suggesting that mitochondrial activity was not upregulated in the Abiraterone Acetate treated cells. Our results suggest the involvement of cytoplasmic reductases, rather than mitochondrial activity, causes the misleading increase in AlamarBlue readings in 2D cultures. While it is common to characterise cellular response to anti-cancer drugs using AlamarBlue^12^, we would caution against using the AlamarBlue assay to study responses to Abiraterone Acetate or Enzalutamide treatment. To our knowledge, the unexpected increase in AlamarBlue signal in parallel with Abiraterone Acetate or Enzalutamide mediated PCa cell death has not been previously reported.

Overall, our results indicate that 3D micro-tumour cultures are less sensitive, than 2D cultures, to changes in androgen signalling introduced either through depletion of androgen from the medium (CSS) or through pharmaceutical inhibition of CYP17A (Abiraterone Acetate). With Docetaxel treatment, the behaviour of the 3D micro-tumours was also markedly different from that seen in the 2D monolayers. The cytotoxic drug family of taxanes, including Docetaxel, target proliferating cells by stabilizing microtubules causing a cell cycle arrest and apoptosis^33^. Therefore, it was expected to observe a considerable difference in response in the slow proliferating 3D micro-tumours compared to more proliferative traditional 2D cultures.

In the present study we demonstrate the utility of the Microwell-mesh as a viable high throughput platform for 3D cancer cell culture and drug screening. A limitation with the Microwell-mesh is that it must be manually fabricated. However, we do provide extensive details here and in our previous publications on the fabrication methods^11,19^. Once a microwell mould has been fabricated, it is relatively easy and inexpensive to generate hundreds of microwell inserts. PDMS is inexpensive, and 500 grams of PDMS can be purchased for ~$100 USD. This is theoretically sufficient to generate 2500 inserts (~0.2 grams/insert), although casting inefficiencies are likely to reduce actual yield to ~1000 inserts. With practice, mesh can be bonded to the microwells efficiently yielding many microwell-mesh well plates in a day. It is not currently possible to purchase the Microwell-mesh platform from a commercial vendor, but growth in the 3D cell culture field may motivate a vendor to consider manufacture of a similar product, which could facilitate quality control, and standardization across the field. Standardized products can have significant commercial value, and this value has motivated the establishment of companies such as QGel (Switzerland) that mass-produces hydrogels that can be used to culture cell spheroids, and multiwell plate products like InSphero GravityTRAP (InSphero, Switzerland) purpose built for spheroid culture. StemCell Technologies (Canada) already manufactures a microwell product (the Aggrewell^TM^) that shares many common features with our Microwell-mesh, and the addition of a mesh to the Aggrewell platform might be a logical next step in StemCell Technologies’ manufacturing process. In the absence of a commercial source, laboratories experienced with the fabrication of microdevices from PDMS will find fabrication of the Microwell-mesh to be a straightforward process that can be rapidly optimised.

Metabolic activity and many other characteristics of 3D micro-tumour cultures can be assessed directly within the Microwell-mesh, or simply removing the mesh allows direct access to hundreds of replicate micro-tumours for histology or other analysis methods such as flow cytometry. Spheroids produced in other platforms (gels or microfluidics) are not as readily accessible^34^. Relative to microfluidics devices, the Microwell-mesh is inexpensive to fabricate, and requires no complex pumps or other equipment to operate. Unlike many gel systems, it is unnecessary to expose cells to UV light to encapsulate them and to use enzyme to later recover/harvest cells from the gel^35,36^. Future work might include the assembly of complex micro-tumours from combinations of PCa and stromal cells to better mimic the bone microenvironment commonly targeted by metastatic PCa^37,38^. Micro-tumours assembled from PCa and bone cells are likely to provide an excellent model with which to screen drugs and to study cell-cell interactions that promote metastasis and/or contribute to drug resistance. In either application, multiple medium exchanges will likely be required. The retention of micro-tumours within discrete microwells in the Microwell-mesh by the nylon mesh is a significant improvement over conventional microwell platforms, extending the utility of the microwell platform to more complex culturing regimes.

## Electronic supplementary material

Supplementary Information
