# Supplementary material for: The Microwell-mesh: A high-throughput 3D prostate cancer spheroid and drug-testing platform
Source: Sci Rep. 2018 Jan 10;8:253. doi: 10.1038/s41598-017-18050-1 (PMC5762676; doi:10.1038/s41598-017-18050-1)
Supplement: Supplementary file 1 — Supplementary Information [file 41598_2017_18050_MOESM1_ESM.pdf]

The Microwell-mesh: A high-throughput 3D prostate cancer spheroid and drug-testing platform

Mosaad, E.O.[1-3]; Chambers, K.F.[4]; Futrega, K.[1]; Clements, J.A.[2]; Doran, M.R[1, 2, 5*].

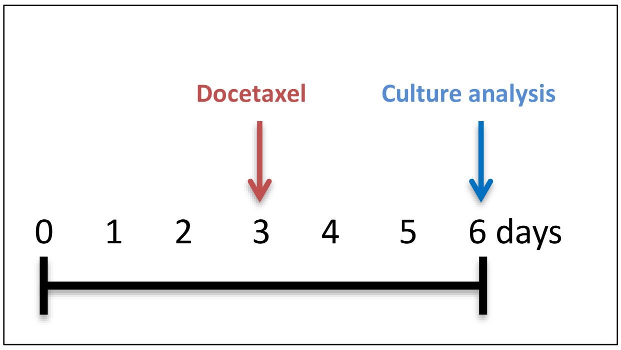

Supplementary Figure 1: Single Docetaxel treatment protocols of prolonged time cultures. A single treatment of Docetaxel was performed after 72 hours of seeding the cells. Drug response was then assessed following three days of exposure to Docetaxel (day 6).

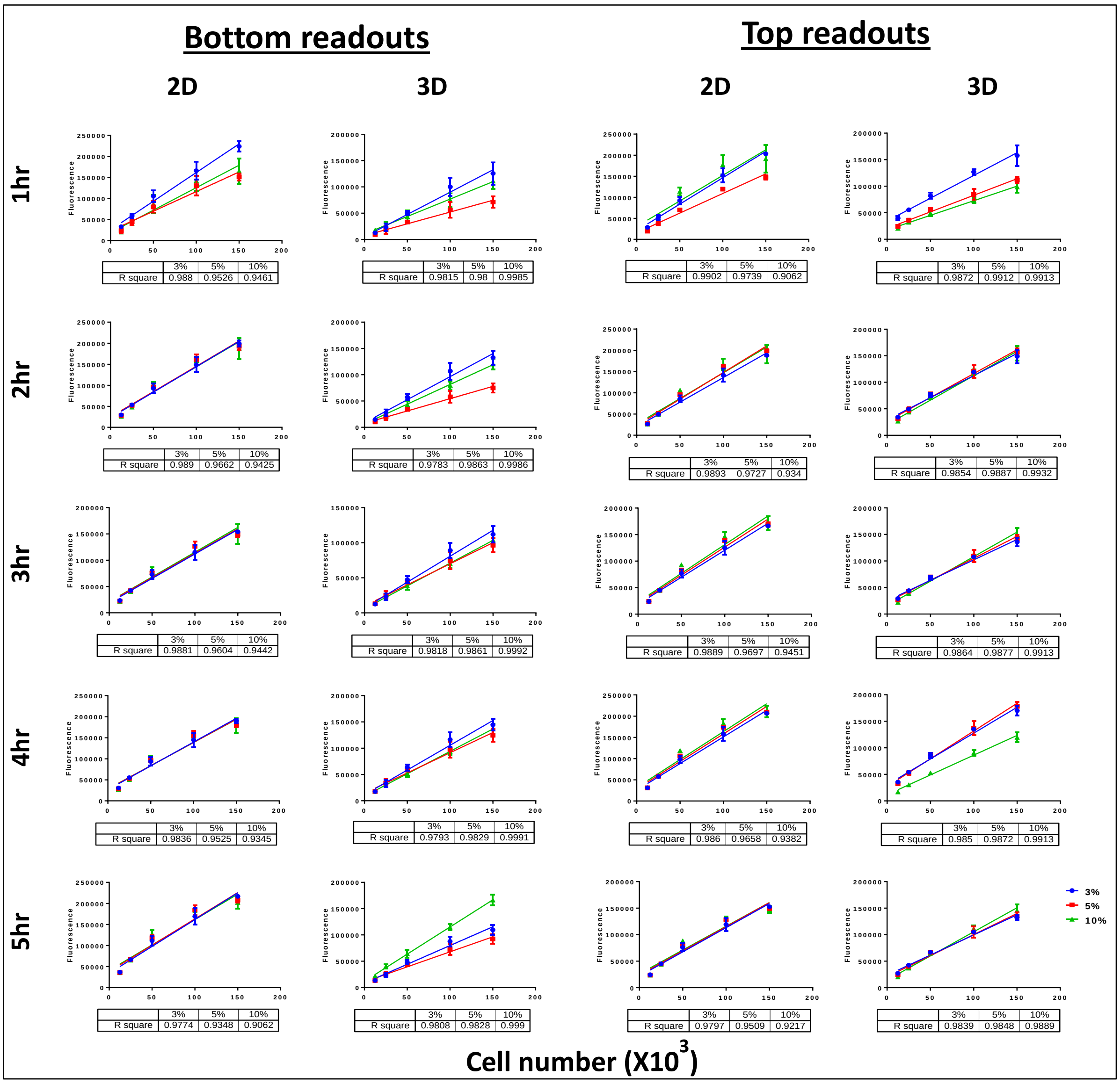

Supplementary Figure 2: AlamarBlue assay optimization in 48-well plate. Cells were seeded overnight in 2D and 3D cultures at the indicated cell densities (12.5, 25, 50, 100 and 150x10^3 cells/well). AlamarBlue reagent was added in three different concentrations (3, 5 and 10%). The fluorescence readouts (nm) were acquired after 1, 2, 3, 4 and 5 hours of incubation. At every time point, the fluorescence was measured from the top and the bottom of the plate. The fluorescence values were plotted against cell numbers to determine the linearity of the assay. R square values of the three tested AlamarBlue concentrations (3, 5 and 10%) are represented under each graph.

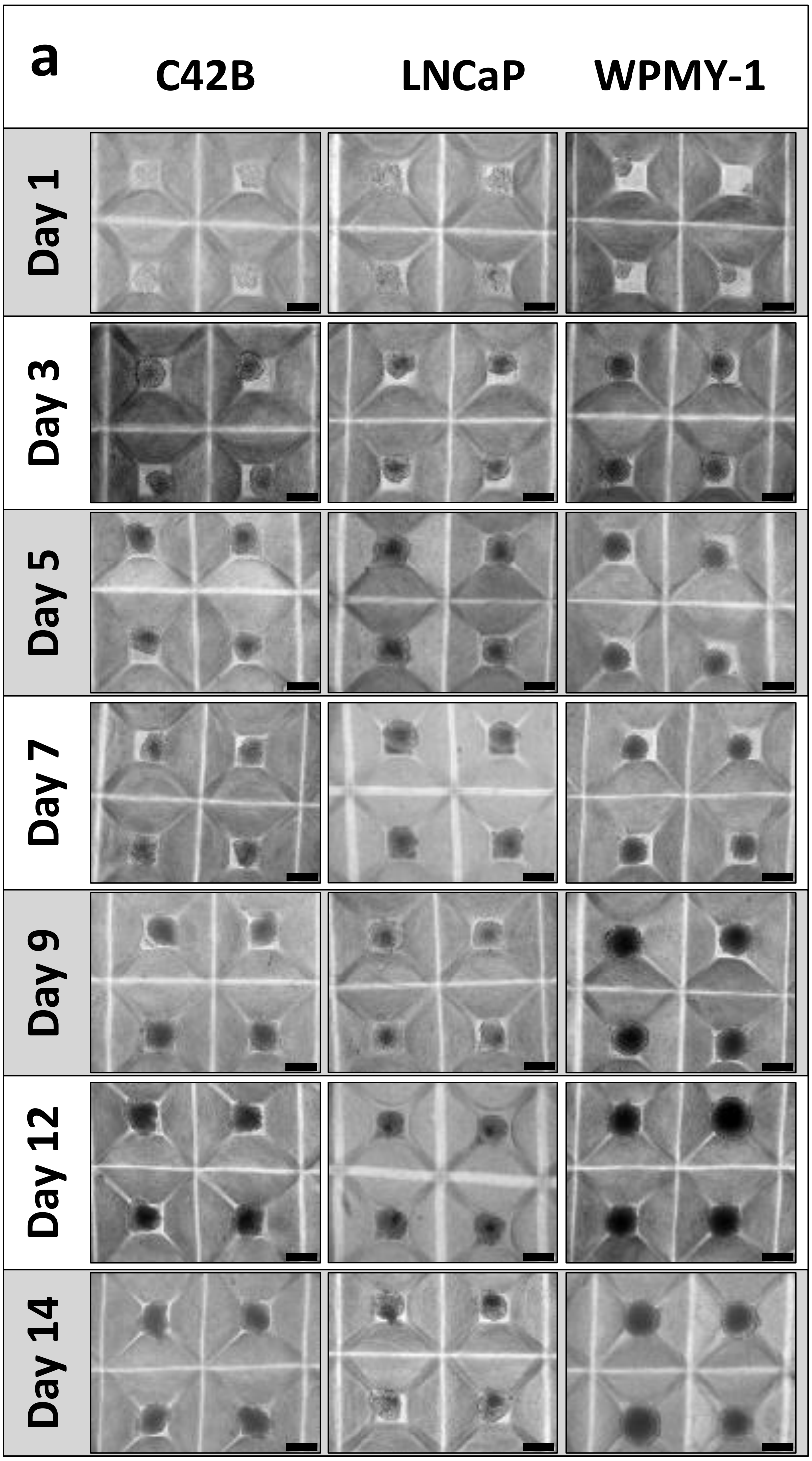
a
C42B
LNCaP
WPMY-1
Day 1
Day 3
Day 5
Day 7
Day 9
Day 12
Day 14

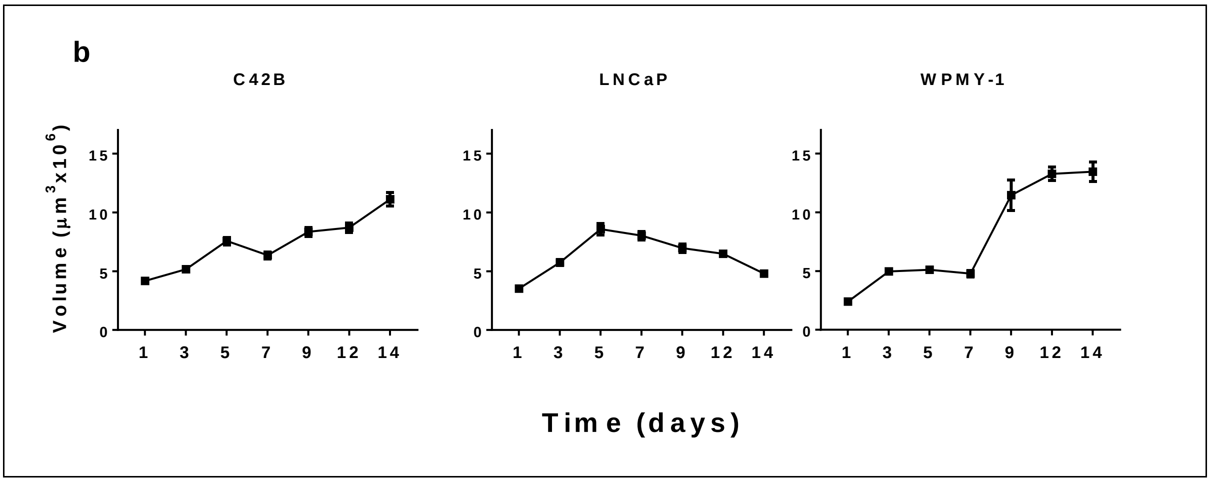

Supplementary Figure 3: Prostate cancer cell line in 3D micro-tumour culture. Prostate cancer (C42B and LNCaP) and prostate myofibroblasts (WPMY-1) cell lines were cultured in the 3D platform (600 cells/spheroid). **(a)** Bright field images were captured every two days. **(b)** Spheroids volume was calculated via diameter measurement of 50 spheroids every two days (mean ± SD). Scale bar = 200 µm.

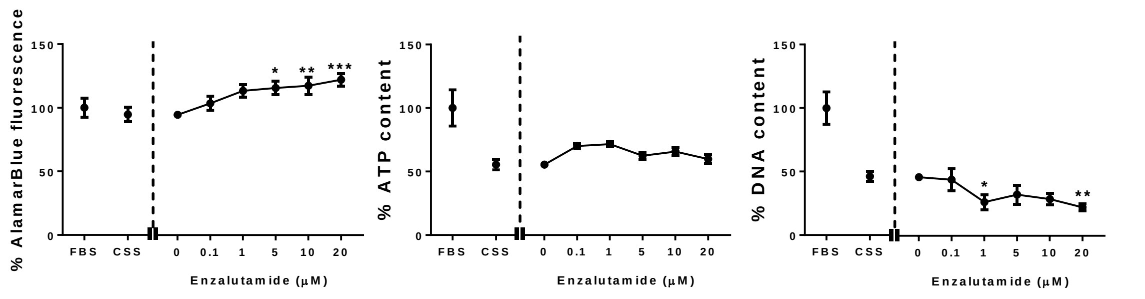

Supplementary Figure 4: Monolayer behaviour of LNCaP cells in androgen deprived conditions. LNCaP cells were seeded in expansion culture medium for 24 hours followed by medium exchange to androgen-depleted medium (CSS) for a further 48 hours. Enzalutamide was then added to the culture medium at the indicated concentrations for an additional 48 hours. AlamarBlue fluorescence, ATP quantity and DNA quantity were then assessed. All results are represented as a percentage of the FBS-containing culture medium control values (Three independent experiments each had four replicate cultures).Statistical significance was calculated by two-way ANOVA compared to the corresponding zero value (*** P<0.001, ** P<0.01 and * P<0.05).

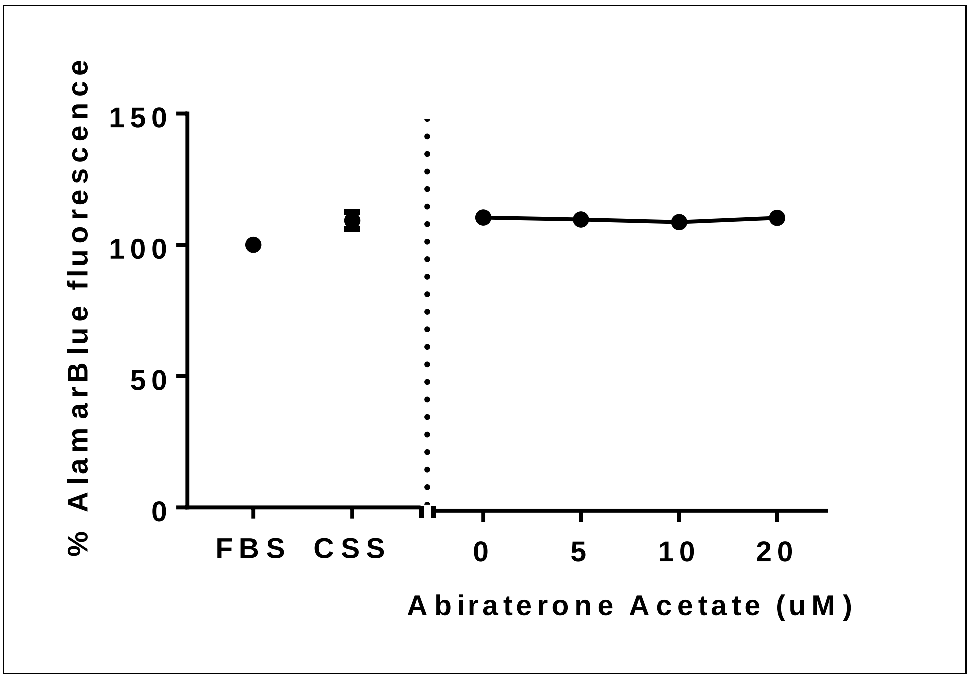

Supplementary Figure 5: AlamarBlue assay of cell-free cultures. In the absence of cancer cells, AlamarBlue signal unchanged with the serial concentrations of the antiandrogen drug, Abiraterone Acetate.

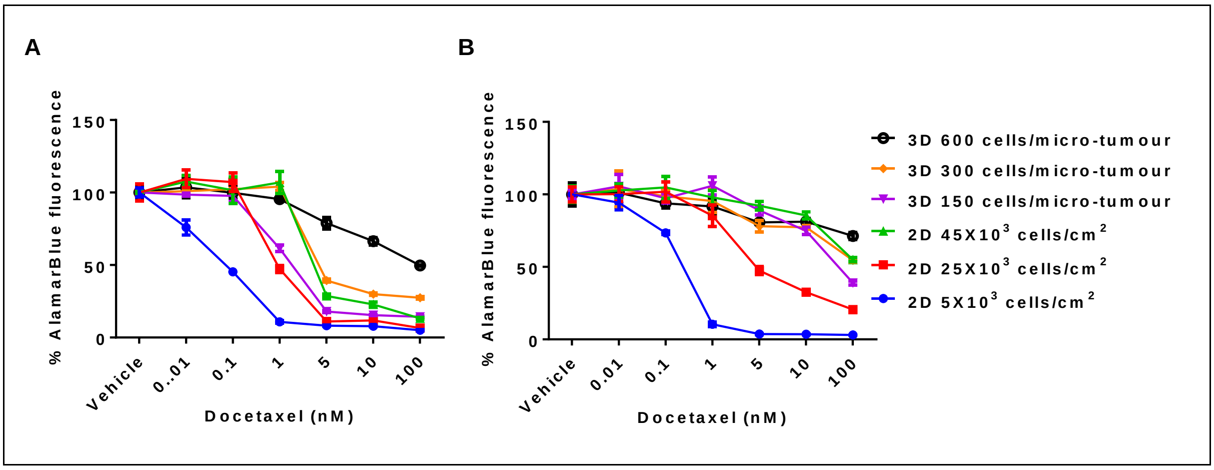

Supplementary Figure 6: Cell culture density impact on *in vitro* cytotoxic drug response. Cells were grown in 2D and 3D cultures in the indicated densities following the single Docetaxel treatment protocols in Figure 1B and Supplementary Figure 1. Compared to 1 day culture prior drug treatment **(A)**, the cultures with prolonged period (3 days) prior Docetaxel treatment **(B)** are less sensitive to the cytotoxic drug.
